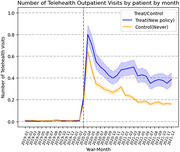# The Evaluation of Telehealth Provider Payment Parity on Health Service Utilization among Adults with High Risk Factors for Dementia in the US

**DOI:** 10.1002/alz.094309

**Published:** 2025-01-09

**Authors:** Zhang Zhang

**Affiliations:** ^1^ University of North Carolina at Chapel Hill, Chapel Hill, NC USA

## Abstract

**Background:**

Cardiometabolic diseases and mental health disorders, which are high‐risk factors for dementia and cognitive decline, are associated with higher mortality and morbidity with age. Interventions before age 60 may lessen the burden of cognitive and physical function in later life. Telehealth offers early intervention and solutions for their complex demands in continuous behavior monitoring and medication refilling. Telehealth has been catalyzed by the COVID‐19 pandemic and become a new norm in healthcare. One of the important factors driving this transformation is governments' temporary modifications and policies. Our study aims to evaluate the impact of telehealth payment parity mandates, which require an equal payment rate for telehealth vs. in‐person outpatient visits, on overall health service utilization among populations with high‐risk factors for dementia.

**Method:**

Using the IBM MarketScan dataset from 2019 to 2021, we leverage a policy shock using a quasi‐experiment by a two‐way fixed effect difference‐in‐differences (DiD) estimation. Our sample includes adults with cardiometabolic risks (diabetes, hypertension, or dyslipidemia) or mental health disorders. We compare the differences between the control and treated states in changes in the outcomes of interest after the onset of the pandemic.

**Result:**

On average, the treated group had 0.18 more telehealth visits by patient by month than the control (p<0.05). Compared to the control group, the treated group had 7 percentage points higher probability of having at least one telehealth encounter by patient by month (p<0.1) and 5 percentage points increase in the proportion of outpatient visits via telehealth by patient by month (p<0.1). In addition, the payment parity did not lead to a significant increase in in‐person outpatient visits.

**Conclusion:**

Our results suggested that the telehealth payment parity, driven by supply‐side incentives, has effectively increased telehealth adoption during and post‐pandemic. The findings addressed the public concerns that telehealth expansion policies could induce and trigger excess demand for in‐person office visits. Our study provides evidence for policymakers to establish sustainable telehealth reimbursement policies and payment models and improve equitable telehealth access for populations with high‐risk factors for dementia in the US.